# Comparative Bioinformatic Analysis of the Proteomes of Rabbit and Human Sex Chromosomes

**DOI:** 10.3390/ani14020217

**Published:** 2024-01-09

**Authors:** Patrícia Pinto-Pinho, João Soares, Pedro Esteves, Rosário Pinto-Leite, Margarida Fardilha, Bruno Colaço

**Affiliations:** 1 Centre for the Research and Technology of Agro-Environmental and Biological Sciences, University of Trás-os-Montes and Alto Douro, 5000-801 Vila Real, Portugal; bcolaco@utad.pt; 2 Laboratory of Signal Transduction, Institute of Biomedicine, Department of Medical Sciences, University of Aveiro, 3810-193 Aveiro, Portugal; mfardilha@ua.pt; 3 Laboratory of Genetics and Andrology, Hospital Center of Trás-os-Montes and Alto Douro, E.P.E., 5000-508 Vila Real, Portugal; mlleite@chtmad.min-saude.pt; 4 Experimental Pathology and Therapeutics Group, IPO Porto Research Center, Portuguese Institute of Oncology of Porto Francisco Gentil, E.P.E., 4200-072 Porto, Portugal; 5 Department of Computer Science, Faculty of Sciences, University of Porto, 4169-007 Porto, Portugal; joao.soares@fc.up.pt (J.S.); pjesteves@cibio.up.pt (P.E.); 6 Center for Research in Advanced Computing Systems, Institute for Systems and Computer Engineering, Technology and Science (CRACS—INESC TEC), 4150-179 Porto, Portugal; 7 Department of Biology, Faculty of Sciences, University of Porto, 4169-007 Porto, Portugal; 8 CIBIO—Research Centre in Biodiversity and Genetic Resources, InBIO Associate Laboratory, 4485-661 Vairão, Portugal; 9 BIOPOLIS Program in Genomics, Biodiversity and Land Planning, Research Centre in Biodiversity and Genetic Resources, 4485-661 Vairão, Portugal; 10 Animal and Veterinary Research Centre, University of Trás-os-Montes and Alto Douro, 5001-801 Vila Real, Portugal

**Keywords:** proteomics, rabbit, sex chromosomes, sex-specific proteins, bioinformatics

## Abstract

**Simple Summary:**

Due to limited proteomic data for rabbit spermatozoa and less comprehensive databases compared to humans, we conducted a combined bioinformatic analysis of the proteome of rabbit X (RX) and human X and Y (HX and HY) chromosomes to identify membrane-associated proteins, particularly those accessible from the cell surface, for potential applications in sperm sexing techniques. Our analysis found 100 (RX), 211 (HX), and 3 (HY) plasma membrane or cell surface-associated proteins, of which 61, 132, and 3 are potentially accessible from the cell surface. Notably, among the HX targets, 60 could serve as additional RX targets not previously identified, bringing the total to 121 RX targets. Furthermore, at least 53 out of the 114 potential common HX and RX targets chromosomes have been previously identified in human spermatozoa, emphasizing their potential as targets of X-chromosome-bearing spermatozoa. The utility of these proteins as targets of rabbit X-chromosome-bearing spermatozoa warrants further exploration.

**Abstract:**

Studying proteins associated with sex chromosomes can provide insights into sex-specific proteins. Membrane proteins accessible through the cell surface may serve as excellent targets for diagnostic, therapeutic, or even technological purposes, such as sperm sexing technologies. In this context, proteins encoded by sex chromosomes have the potential to become targets for X- or Y-chromosome-bearing spermatozoa. Due to the limited availability of proteomic studies on rabbit spermatozoa and poorly annotated databases for rabbits compared to humans, a bioinformatic analysis of the available rabbit X chromosome proteome (RX), as well as the human X (HX) and Y (HY) chromosomes proteome, was conducted to identify potential targets that could be accessible from the cell surface and predict which of the potential targets identified in humans might also exist in rabbits. We identified 100, 211, and 3 proteins associated with the plasma membrane or cell surface for RX, HX, and HY, respectively, of which 61, 132, and 3 proteins exhibit potential as targets as they were predicted to be accessible from the cell surface. Cross-referencing the potential HX targets with the rabbit proteome revealed an additional 60 proteins with the potential to be RX targets, resulting in a total of 121 potential RX targets. In addition, at least 53 possible common HX and RX targets have been previously identified in human spermatozoa, emphasizing their potential as targets of X-chromosome-bearing spermatozoa. Further proteomic studies on rabbit sperm will be essential to identify and validate the usefulness of these proteins for application in rabbit sperm sorting techniques as targets of X-chromosome-bearing spermatozoa.

## 1. Introduction

Understanding the proteins encoded by the X and Y chromosomes (X-proteins and Y-proteins) is crucial for gaining a better insight into overall health status since sex chromosomes play a role beyond reproductive functions, namely in immune responses and defenses, predisposition and pathogenesis of neurodegenerative disorders, or even obesity and metabolic disorders [1,2,3,4,5,6]. This knowledge is also important for uncovering novel biomarkers for sex-linked diseases and practical applications, such as the manipulation and separation of spermatozoa based on their sex chromosomes [7,8]. This latter application stands out as highly valuable for implementation in animal breeding farms where one of the sexes may be preferred over the other. In those cases, the sex of the offspring may directly affect the profits and sustainability of the production lines. Having a sperm sexing technique that would allow for the selection of the desired offspring before they are born is a valuable tool, already commercially available for some species, but mainly implemented for cattle (reviewed in [9]).

Cuniculture would also benefit from such a technique since female parents and grandparents hold considerably higher value compared to the males born from the same litter [10,11]. These bucks lack the desired traits for both reproduction and efficient meat production and, therefore, translate into financial losses for producers of breeding does. However, up to the present day, no sperm sexing technique that is both sufficiently accurate and economically viable for application in cuniculture has been developed (reviewed in [11]).

Among the most promising technologies for integration into a sexing method for rabbits are immunological methods, relying on targeting proteins specific to the X- or Y-chromosome-bearing spermatozoa (X- or Y-sperm). If there exist genomic DNA variations between X- and Y-sperm, there might be different gene expression and consequently, molecular differences among proteins present on the surface of rabbit X- and Y-sperm [7,12].

Despite the paramount importance of rabbits as animal models in reproductive studies, as well as the significance of comprehending the proteome of spermatozoa for reproductive biology and animal breeding, a search conducted on PubMed for papers available online until 10 August 2023, under the keywords “rabbit”, “semen” or “spermatozoa”, and “proteome” or “proteomics”, revealed a notable gap in the available literature. This search yielded only seven proteomic studies focusing on the rabbit seminal fluid and/or spermatozoa proteome, published between 2014 and 2022 [13,14,15,16,17,18,19]. Furthermore, these studies were primarily focused on the overall proteome without delving into the specific differences between X-proteins and Y-proteins. Moreover, only the proteome of the rabbits’ X chromosome is available in UniProt or NCBI databases [20,21]. The absence of a comprehensive Y chromosome proteome dataset for rabbits undermines the feasibility of determining proteins specific to the rabbit Y-sperm.

On the other hand, the proteome of both human sex chromosomes is available [22]. Since conservation is often observed in protein-coding genes and their functions across species, a combined analysis would allow for a deeper understanding of potentially orthologous proteins in rabbits [23,24].

Therefore, the present study aims to perform a bioinformatic analysis of both rabbit and human sex chromosomes proteomes to ultimately determine which proteins may be localized within the plasma membrane and conveniently accessible from the cell surface, underscoring their potential utility for sperm sexing techniques. The integration of both rabbit and human proteomes also contributes to unveiling possible species-specific and shared targets, envisioning the potential for targets to be translatable across multiple species. 

## 2. Materials and Methods

To identify potential rabbit and human protein candidates for an immunologically based sperm sexing technique, a bioinformatic analysis was performed. For that, the UniProt database, Ensembl BioMart Tool, eggNOG-mapper v.2.1.12 (eggNOG v.5.0), DeepTMHMM v.1.0.24, AmiGO 2 GENEONTOLOGY, and an automated script were used. To this end, the proteome of the rabbit X chromosome, and the human X and Y chromosomes proteome were analyzed. The analysis was not extended to the rabbit Y chromosome, as the Y chromosome proteome is not yet available in the UniProt or NCBI databases. To identify proteins of interest, i.e., those that might be present on the plasma membrane/cell surface, information about their cellular localization retrieved from both the UniProt database and annotations from one-to-one orthology if these had experimental evidence was considered. Nonetheless, to be used as potential targets, the proteins should be accessible from the cell surface and, therefore, preference should be given to cell surface proteins, plasma membrane proteins located on the external side of the membrane or that have a transmembrane domain, or to transmembrane proteins associated with the extracellular space. For this, the analysis was complemented with a software analysis to predict the protein topology and the presence of transmembrane regions in these proteins based on their sequences. 

### 2.1. Datasets Obtention and Filtering

The *Oryctolagus cuniculus* reference proteome (version of 09 2023, UP000001811 [20]), available in the UniProt database, was used to assess the rabbit X chromosome proteome, and the *Homo sapiens* reference proteome (version of 09 2023, UP000005640 [22]), available in the UniProt database, was used to assess the human X and Y chromosomes proteome.

The available rabbit and human proteomes had a high percentage of duplicate entries (39.5% and 62.0%, respectively). Therefore, the list of protein identifiers (IDs) retrieved for the X and Y chromosomes was filtered to ensure that the analysis was based on a less redundant set of protein IDs. Hence, the IDs were mapped using the ID mapping tool of the UniProt database [25] to retrieve the respective Ensembl Transcript IDs if available. The rabbit and human Ensembl Transcript IDs were used in the Ensembl BioMart tool [26] against the Rabbit Genes (OryCun 2.0) and Human Genes (GRCh38.p14) datasets, respectively, to retrieve the corresponding Gene Stable IDs and information regarding which transcripts were considered canonical transcripts [27]. For that, the attributes “Transcript stable ID”, “Gene Stable ID”, and “Ensembl Canonical” were selected. First, entries with a Transcript stable ID but no associated Gene stable ID were eliminated. Since different transcripts can be products of the same gene if there were multiple entries with the same Gene stable ID, priority was given to retaining the transcripts corresponding to the reviewed entry, if available, followed by those corresponding to the Ensembl Canonical entry. Additionally, any duplicated UniProt entries resulting from a single UniProt ID being mapped to multiple transcripts were removed, with preference given to retaining the Ensembl Canonical entry when applicable.

### 2.2. Functional Annotation

The functional annotation of the sequences involved a dual approach, incorporating information from both the UniProt database and data obtained by orthology assignments using the eggNOG-mapper v.2.1.12 tool [28,29,30].

#### 2.2.1. UniProt Database

The ID mapping tool of the UniProt database was used to retrieve the gene name, protein name, length, and Gene Ontology (GO) IDs (representing gene product properties) of each protein. All GO IDs associated with the entries were considered, as only a limited number of entries possessed experimentally verified GO annotations.

#### 2.2.2. eggNOG-Mapper

The eggNOG-mapper tool was used for functional annotation of the datasets of proteins from the X and Y chromosomes based on fast orthology assignments. For annotation, the taxonomic scope was auto-adjusted per query, only annotations from one-to-one orthology were transferred, and GO evidence was only transferred if annotations had experimental evidence. Regarding search filters, the following parameters were used: a minimum hit e-value of 0.001, a minimum hit bit-score of 60, a percentage of identity of 80%, a minimum percentage of query coverage of 80%, and a minimum percentage of subject coverage of 80%. All the other parameters were used according to the tool’s default values. Gene names, a description, and GO IDs were obtained from eggNOG. 

### 2.3. Protein Topology Prediction

The topology of transmembrane proteins and the number of transmembrane regions (TMR) were predicted using the DeepTMHMM v.1.0.24 [31,32]. The DeepTMHMM software encodes a protein sequence to predict its topology based on the correspondent per-residue sequence of labels, using a deep learning encoder–decoder sequence-to-sequence model [32]. To achieve accurate predictions, it combines a large pre-training, a bidirectional long short-term memory that is a neuronal network capable of reading the protein sequence in both forward and backward directions, and a dense layer with drop-out that prevents overfitting, thereby enhancing the overall performance of the model [32,33,34]. After encoding the representations, a conditional random field that enables both the decoding of the most probable sequence and the calculation of marginal probabilities at each position is employed to assign probabilities to the entire output sequence [32]. The DeepTMHMM software was trained and tested using a dataset of 3574 sequences covering five protein types—alpha helical transmembrane proteins without a signal peptide (TM), alpha helical transmembrane proteins with a signal peptide (TM+SP), beta-barrel transmembrane proteins (BETA), globular proteins without a signal peptide (GLOB), and globular proteins with a signal peptide (GLOB+SP). Moreover, to mitigate the risk of over-optimistic assessments, the sequence identity within each type was set at 30 percent [32].

Therefore, upon processing the FASTA file containing the protein sequences from our dataset, sourced from UniProt, the proteins were categorized as TM, BETA, or GLOB, and the presence of a SP was also predicted.

### 2.4. Identification of Proteins Associated with the Plasma Membrane

To identify the key group of proteins relevant to the research, an automated script was used (available at GitHub [35]) to generate a list with the union of the GO IDs obtained from UniProt and eggNOG associated with each protein (determined as previously described in Section 2.2.1 and Section 2.2.2) and classify a protein as “of interest” if any of the following GO IDs of interest were associated to it: GO:0005886 (plasma membrane, PM), GO:0005904 (PM), GO:0009986 (cell surface, CS), GO:0009928 (CS), GO:0009929 (CS), GO:0009897 (external side of plasma membrane, ESPM), GO:0031232 (extrinsic component of external side of plasma membrane, ECESPM), GO:0046658 (anchored component of plasma membrane, ACPM), GO:0031362 (anchored component of external side of plasma membrane, ACESPM), GO:0071575 (integral component of external side of plasma membrane, ICESPM), GO:0005887 (integral component of plasma membrane, ICPM), GO:0010339 (external side of cell wall, ESCW), or GO:0031240 (external side of cell outer membrane, ESCOM). Proteins whose only GO ID of interest was the GO:0005615 for extracellular space (ES) were also considered of interest if they were predicted to be transmembrane proteins according to the DeepTMHMM analysis.

After crossing the list of proteins of interest with the results of DeepTMHMM, a list of possible targets, i.e., proteins possibly accessible from the cell surface, was created by considering all proteins with GO terms for CS, ESPM, ECESPM, ACESPM, ICESPM, ESCW, and/or ESCOM, and all proteins with GO terms for PM, ACPM, ICPM, and/or ES that were predicted to be transmembrane proteins.

All GO terms of interest and respective IDs were selected and retrieved from the AmiGO 2 GENEONTOLOGY web application [36,37,38,39]. The ACPM term was still considered despite it having been considered obsolete on 26 August 2022 since it represents a protein topology and not a cellular component.

Furthermore, it was confirmed which of the obtained potential targets were selected based on GO terms of interest attributed to the entry through experimental evidence, according to UniProt and/or eggNOG annotation.

### 2.5. Statistical Overrepresentation Test of the Rabbit X Chromosome Proteome

To assess whether the set of proteins within the rabbit X chromosome proteome exhibited underrepresented or overrepresented biological processes, molecular functions, cellular components, protein classes, or pathways in comparison to the Reference Proteome Genome list of *Oryctolagus cuniculus*, a statistical overrepresentation test was conducted using PANTHER 18.0 [40]. Three distinct protein lists were analyzed: the complete list of filtered protein entries from the rabbit X chromosome, the list of protein entries from the rabbit X chromosome that were of particular interest (obtained as described in Section 2.4), and the list of potential targets among the protein entries from the rabbit X chromosome, which are potentially accessible from the cell surface (obtained as indicated in Section 2.4). To conduct this analysis, individual text document files (.TXT) containing the respective UniProt IDs were uploaded to the PANTHER 18.0 software, following the methodology of Mi and collaborators [41]. The Fisher’s Exact test with Bonferroni correction for multiple testing was employed and only the significantly overrepresented terms were considered for analysis (*p*-value < 0.05).

### 2.6. Cross-Species Analysis: Identification of Human Targets in the Rabbit Proteome

For this analysis, a set of tools for automatic data extraction, sequence alignment, and similarity study was developed.

#### 2.6.1. Data Extraction

**Figure 1:**
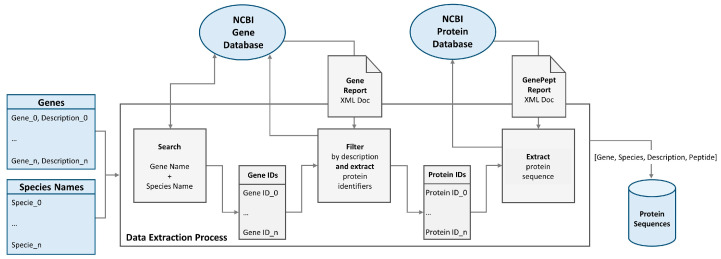
Data extraction pipeline.

For the data extraction process, a tool was designed that, given a set of gene names and a set of species names, downloads the protein sequences of known resulting products for each gene and species combination, as presented in Figure 1.

This tool was implemented in Python and the NCBI database was used as a data source using the Entrez Programming Utilities (E-utilities) API. For each gene name and species name combination, a gene search was conducted on the Gene NCBI database, using the corresponding URL and the respective arguments. This returned the gene identifiers of the different genes. Each gene identifier was filtered based on the given description and the corresponding gene report was downloaded in XML format using the Gene NCBI database. The gene report was then parsed to extract Protein Reference Sequences (RefSeq) identifiers. The filtering process allowed the prevention of different genes with identical names, due to gene aliases, from compromising the final dataset. For each RefSeq identifier, the corresponding GenPept data were downloaded, in XML format, using the Protein NCBI database. From the resulting data, the protein sequence was extracted.

#### 2.6.2. Alignment and Similarity

For the sequence alignment and similarity process, we designed a tool that, given a set of gene names, a set of species names, and a reference species name, aligns the protein sequences and measures their similarity against the given reference. This tool was implemented in Python, using the data resulting from the data extraction process.

For each gene name, the protein sequence from each species (including reference species) was selected. For simplicity, a single sequence was selected for each species, although more than one can exist. When the sequence corresponding to isoform X1 was available, it was selected. Otherwise, isoform 1 was chosen. In the absence of both isoform X1 and isoform 1, the sequence with the greater length was utilized. The sequences were then aligned against the reference sequence, using Python’s Biopython library. For the alignment, a global pairwise alignment strategy was selected using the match score and gap penalty parameters similar to the ones used in NCBI’s Protein Blast tool (i.e., blosum62 matrix, −11, −1 for matching and mismatching, gap opening, and gap extension, respectively).

The similarity score was calculated by counting the number of equal pairs of the aligned sequences and dividing it by the difference between the length and the number of indels of the aligned sequence.

#### 2.6.3. Manual Curation

All human protein targets of the X chromosome (X-targets) that were not identified in rabbits were manually checked, as they could have an entry with an LOC gene ID or no gene name associated, which would prevent the script from selecting it. In this case, the percentage of similarity between the rabbit and human protein sequences was verified, as well as whether the respective entries were identified as orthologs in the NCBI database. If orthology was confirmed, it was checked whether it was a target previously identified for rabbits (and therefore common) or if it was a potential additional X-target for rabbits. The human X-targets that, according to the script, had not been identified as rabbit X-targets in the initial analysis were also analyzed, giving special attention to proteins whose sequence similarity percentage was less than 70% [42,43,44]. Among these, we eliminated proteins whose entries had been removed from the NCBI database due to standard genome annotation processing, proteins that, while not being unplaced in the rabbit proteome, were encoded by a chromosome other than the X, proteins that had been mapped to the same NCBI rabbit protein entry (keeping, in this case, the entry whose gene of interest corresponded to that of the entry as the main gene and not as an alias), and proteins for which the rabbit entry was not identified as an ortholog of the human entry, according to the NCBI database (unless they had identical neighboring genes). Furthermore, some of the human X-targets identified by the script as possible extra targets for rabbits could correspond to one of the rabbit entries that did not have a gene/protein name according to UniProt. Therefore, for all proteins without a specific gene or protein name, the names assigned by the annotation performed with eggNOG were cross-referenced with the list of possible extra targets to identify potential matches. When a match was found, the percentage of sequence similarity and the orthology between humans and rabbits were verified. If positive, it was confirmed whether the protein had already been identified as a rabbit X-target and, therefore, was a common target, or if it was a possible additional rabbit X-target.

### 2.7. Cross-Reference of the Common Human and Rabbit Targets with Human Spermatozoa Proteins

To further understand which of the possible common human and rabbit targets (as obtained from the analysis described in Section 2.6) have already been described in human spermatozoa, the list of targets was cross-referenced based on the gene name and/or UniProt entry ID with a list of proteins identified in human spermatozoa, previously compiled by our research group [45]. Briefly, to obtain this list of human spermatozoa proteins, a literature search was conducted on PubMed, Scopus, and Web of Science databases until 23 January 2023, using the terms “sperm” or “spermatozoa” or “spermatozoon”, and “proteomics” or “proteome” or “protein profile” or “proteomic analysis”, and “human” or “*Homo sapiens*”. Only English and Portuguese studies focusing on human-ejaculated spermatozoa and disclosing a UniProtKB/Swiss-Prot ID or gene name for each protein were considered. 

## 3. Results

### 3.1. Proteomes Filtering Process

**Figure 2:**
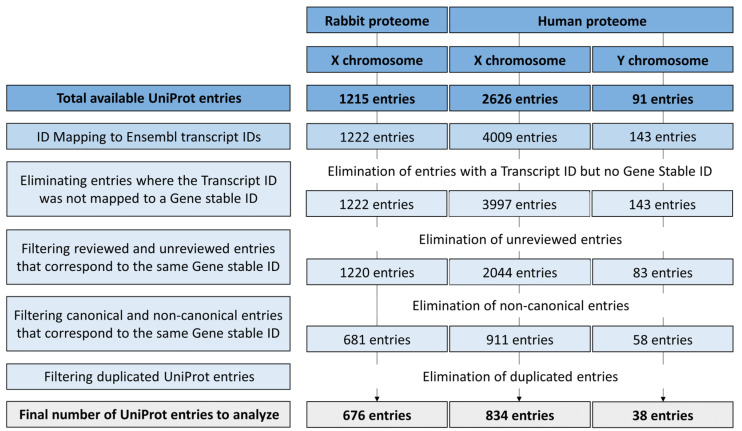
Flow diagram of the filtering process of the rabbit X chromosome proteome and human X and Y chromosome proteomes to generate a less redundant set of protein IDs for subsequent analysis.

A total of 1215 ID entries that are associated with the rabbit X chromosome were retrieved from UniProt, comprising 1222 (unique) transcript stable IDs and 1222 gene stable IDs (681 unique). After filtering, a total of 676 UniProt entries remained for further analysis (Figure 2).

The same filtering process was performed for the human X and Y chromosomes. Out of 2626 entries for the human X chromosome, 9 were unmapped and the others were mapped to a total of 4000 transcript stable IDs (3951 unique) and 3988 gene stable IDs (864 unique). Regarding the 91 entries of the human Y chromosome, 1 ID was unmapped and the others were mapped to 142 (unique) transcript stable IDs and 142 gene stable IDs (45 unique). Following filtering, a total of 834 and 38 UniProt entries were retained for further analysis for the X and Y chromosomes, respectively (Figure 2). 

### 3.2. Analysis of the Sex Chromosomes Proteome

**Figure 3:**
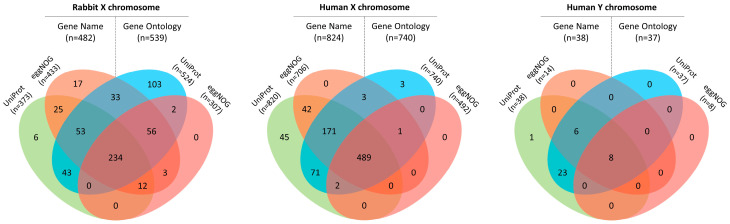
Venn diagrams illustrating the number of protein entries selected for analysis from the rabbit X chromosome proteome (676 entries) and the human X (834 entries) and Y (38 entries) chromosome proteomes that contain information on gene name and/or Gene Ontology information either obtained directly from UniProt or transferred annotations from one-to-one orthology after functional annotation prediction using eggNOG-mapper v.2.1.12. These diagrams provide insights into the commonalities and distinctions in protein annotation from different information sources, highlighting their complementary nature.

By integrating UniProt information for each protein entry with eggNOG’s functional annotation through fast orthology assignment, a more comprehensive characterization of the selected proteins for further analysis was achieved, particularly in terms of gene names and GO information (Figure 3).

#### 3.2.1. Characterization of the Rabbit X Chromosome Proteome

By retrieving GO information from UniProt (n = 524, 77.5%) and eggNOG (n = 307, 45.4%), it was possible to annotate 539 rabbit protein entries (79.7%). Of these, a total of 108 entries (UniProt, n = 65; eggNOG, n = 87) had GO IDs that could indicate a possible cellular localization of interest (Figure 3). This information was complemented with the topology prediction performed with the deepTMHMM v.1.0.24 software. In total, 519 proteins were predicted to be of the globular type, while 130 were predicted to be alpha-helical transmembrane proteins, and 27 proteins were exclusively predicted to have a signal peptide. Among the transmembrane proteins, 29 were also predicted to have an SP and the transmembrane regions ranged from 1 to 18.

By combining all available data, it was possible to identify a group of 100 proteins of interest that may be present in the plasma membrane, from which 61 are possibly accessible from the cell surface (rabbit X-targets) (Table 1; Supplementary Figure S1; Supplementary Spreadsheet S1). These proteins represent a group of particular interest for further investigation, due to their potential use as protein targets for sperm sexing. Out of the 61 possible targets, 46 have been selected based on experimentally validated GO information, although not necessarily in spermatozoa. 

##### Overrepresentation Analysis of the Rabbit X Chromosome Proteome

**Figure 4:**
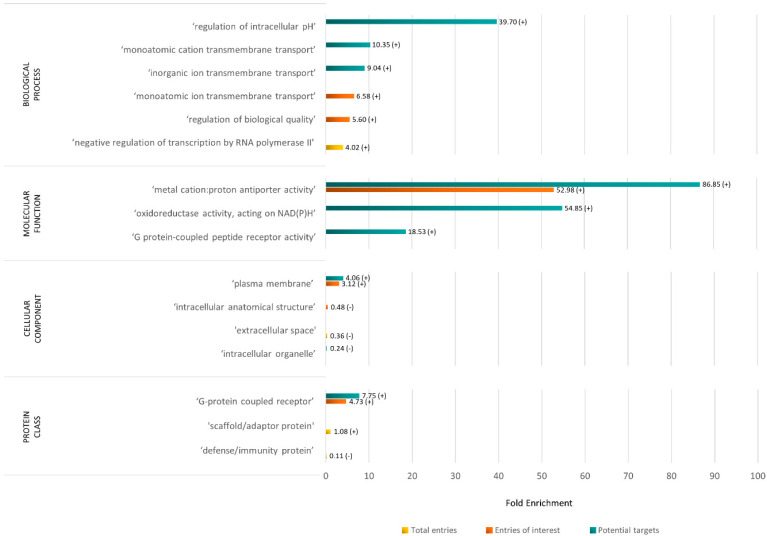
Summary of the results of the statistical overrepresentation test performed with PANTHER 18.0 for the total list of proteins of the rabbit X chromosome under analysis (in yellow, 646 entries mapped out of 676), the list of protein entries of interest (in orange, 99 entries mapped out of 100), and the list of potential targets (in blue, 60 entries mapped out of 61). The fold enrichment of each significantly over-represented (+) or under-represented (−) term by category is shown (*p*-value < 0.05, Fisher’s exact test, Bonferroni correction for multiple testing).

To gain a deeper understanding of the potential enrichments in terms of biological processes, molecular functions, cellular components, protein classes, and pathways within the rabbit X chromosome and the obtained sub-lists compared to the Reference Proteome Genome of *Oryctolagus cuniculus*, an overrepresentation analysis was conducted using PANTHER 18.0 (results summarized in Figure 4).

Among the total 676 entries subjected to analysis, 646 were successfully mapped. The analysis revealed a significant overrepresentation of the biological process ‘negative regulation of transcription by RNA polymerase II’ (Fold Enrichment [FE] = 4.02) and an underrepresentation of the cellular component ‘extracellular space’ (FE = 0.36). Additionally, the ‘scaffold/adaptor protein’ class was found to be overrepresented (FE = 1.08), whereas the ‘defense/immunity protein’ class showed underrepresentation (FE = 0.11).

When focusing on the set of 100 entries of interest, 99 were mapped and revealed an overrepresentation of the biological processes ‘monoatomic ion transmembrane transport’ (FE = 6.58) and ‘regulation of biological quality’ (FE = 5.60). The molecular function ‘metal cation:proton antiporter activity’ was also overrepresented (FE = 52.98). As expected, the cellular component ‘plasma membrane’ showed to be overrepresented (FE = 3.12), while the ‘intracellular anatomical structure’ was found to be underrepresented (FE = 0.48). Moreover, the protein class ‘G-protein coupled receptor’ was overrepresented (FE = 4.73). 

Finally, the list of potential targets was assessed, and 60 out of the 61 entries were successfully mapped. The analysis revealed an overrepresentation of the biological processes ‘regulation of intracellular pH’ (FE = 39.70), ‘monoatomic cation transmembrane transport’ (FE = 10.35), and ‘inorganic ion transmembrane transport’ (FE = 9.04), as well as of the molecular functions ‘metal cation:proton antiporter activity’ (FE = 86.85), ‘oxidoreductase activity, acting on NAD(P)H’ (FE = 54.85), and ‘G protein-coupled peptide receptor activity’ (FE = 18.53). Once more, the cellular component ‘plasma membrane’ was found to be overrepresented (FE = 4.06), while the cellular component ‘intracellular organelle’ was underrepresented (FE = 0.24). Consistent with the findings from the list of proteins of interest, the protein class ‘G-protein coupled receptor’ was overrepresented (FE = 7.75).

It is worth noting that no pathway was found to be significantly over/underrepresented in any of the protein sets analyzed.

#### 3.2.2. Characterization of the Human X and Y Chromosomes Proteome

GO information was extracted from UniProt for human X and Y chromosome proteins, resulting in annotations for 740 (X, 88.7%) and 37 (Y, 97.4%) protein entries. Additionally, data were obtained from eggNOG, yielding GO annotations for 492 (X, 59.0%) and 8 (Y, 21.1%) protein entries. Therefore, a total of 740 (X, 88.7%) and 37 (Y, 97.4%) protein entries were annotated with GO IDs, respectively (Figure 3). Of these, 231 (X; UniProt, n = 212; eggNOG, n = 129) and 3 (Y; UniProt, n = 2; eggNOG, n = 1) had GO IDs indicating a cellular localization of interest. Regarding the topology prediction of the X and Y chromosome proteins, 619 (X) and 35 (Y) were predicted to be globular proteins, 177 (X) and 2 (Y) alpha-helical transmembrane proteins, and 38 (X) and 1 (Y) were exclusively predicted to have a signal peptide. Within the set of transmembrane proteins, 56 (X) and 2 (Y) were further anticipated to have a signal peptide and the transmembrane regions ranged between 1–24 (X) and 1 (Y).

The consolidation of all available data regarding the human X chromosome permitted the identification of a cluster of 211 proteins associated with the plasma membrane, among which 132 are potentially accessible from the cell surface (human X-targets) (Table 2; Supplementary Figure S1; Supplementary Spreadsheet S1). Out of the 132 possible targets, 75 have been selected based on experimentally validated GO information, although not necessarily in spermatozoa. 

Regarding the human Y chromosome proteome, three proteins were associated with the plasma membrane and all of them are potentially accessible from the cell surface (human Y-targets) (Table 3; Supplementary Figure S1; Supplementary Spreadsheet S1). One of these three possible targets has been selected based on experimentally validated GO information, although not necessarily in spermatozoa.

### 3.3. Cross-Species Analysis: Identification of Human Targets in the Rabbit Proteome

**Figure 5:**
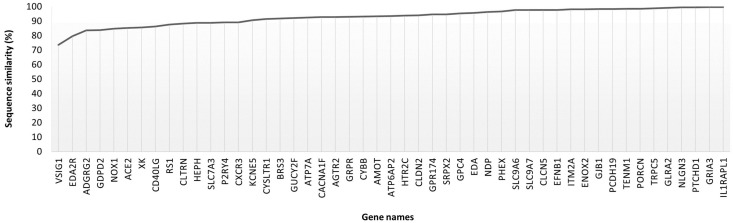
Protein sequence similarity (%) among the 49 human targets that were identified in the list of rabbit targets and the respective rabbit protein with the same gene and protein name.

A script was used to identify rabbit protein entries with gene and protein names similar to the selected human targets, followed by the determination of the percentage of sequence similarity between the two species. Of the 132 human targets, only 130 had a gene and protein name associated according to UniProt, and the script was able to identify 118 of them in the rabbit proteome. A total of 49 proteins were common to the list of rabbit X-targets with known gene names, sharing more than 70% similarity with the human protein sequences (Figure 5). 

Moreover, of the 12 human X-targets with a gene name that were not mapped to the rabbit proteome by the script, it was possible to identify 2 that can be present in the rabbit proteome and specifically encoded by the X chromosome. Those are the olfactory receptor 13H1 (*OR13H1*) and the P2Y receptor family member 10 (*P2RY10*). The olfactory receptor 13H1 is a characterized rabbit and human X chromosome protein. The respective entry was selected as a possible target both in rabbits and humans. Nevertheless, the gene symbol associated with the protein RefSeq available in the NCBI database for the rabbit olfactory receptor 13H1 is LOC100350442, which prevented the script from selecting it as a common human and rabbit target. Both human and rabbit entries are part of the list of *OR13H1* orthologs from NCBI, and the rabbit sequence from the UniProt entry shares 100% and 82.8% similarity with the rabbit (XP_051682900) and human (NP_001004486.1) protein sequences identified in NCBI, respectively. The rabbit protein encoded by *P2RY10* is considered one of the proteins of interest of the rabbit X chromosome but is not in the list of human X-targets identified in the rabbit proteome for similar reasons to the olfactory receptor 13H1; the NCBI entry that shares 100% similarity with the UniProt sequence (XP_008271184, putative P2Y receptor family member 10) has associated the gene symbol LOC100339772. Also, this rabbit protein sequence shares 87.9% similarity with isoform 1 of the protein encoded by the human *P2RY10* (XP_047297954.1), available in the NCBI database. Moreover, if looking at the set of three genes that come immediately before and after the human and rabbit *P2RY10*, it is possible to observe that both are preceded by *LPAR4* and succeeded by *GPR174*. Therefore, there is a possibility that this protein could be an extra rabbit target. Additionally, the human target toll-like receptor 8 (*TLR8*) is known to be expressed in rabbits. Nonetheless, the script was not able to detect any protein RefSeq for rabbits, because the NCBI entry only appears to be associated with the ID AGN12838.1. Yet, in rabbits, the protein is known to be encoded by chromosome 13 [46,47].

**Figure 6:**
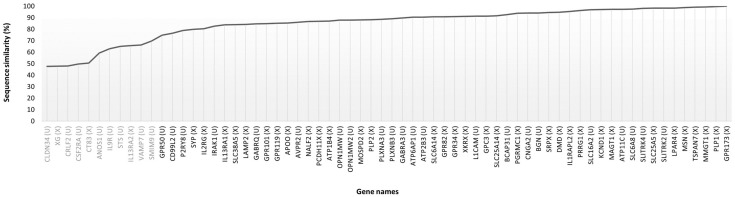
Protein sequence similarity (%) among 66 human X-targets identified in the rabbit proteome (which were not a priori present or characterized in the list of rabbit X-targets) and the corresponding rabbit proteins with the same gene and protein names. Gene names encoding proteins with less than 70% similarity between humans and rabbits are highlighted in grey. The chromosome associated with each rabbit protein is specified in parentheses next to the gene name—(X) X chromosome and (U) unplaced.

Additionally, according to the script results, 69 out of the 118 human targets sharing similar gene and protein names with entries of the rabbit proteome were not included or characterized in the list of rabbit targets. Of these 69 entries, 2 were not further explored since they were removed from NCBI because of standard genome annotation processing. These entries were the predicted transmembrane protein 47 (*TMEM47*) and the neuronal membrane glycoprotein M6-b (*GPM6B*). Moreover, one of the identified proteins, interleukin-3 receptor subunit alpha (*IL3RA*), is encoded by the X chromosome in humans, while in rabbits, it is known to be encoded by chromosome 2 [48]. Therefore, this protein was not considered as a potential extra rabbit X-target. This led to a list of 66 protein entries that were further explored due to their potential to be extra rabbit X-targets; 36 proteins were known to be encoded by the rabbit X chromosome and another 30 were still unplaced (Figure 6). More than 80% (n = 55) of these proteins share more than 70% similarity with the rabbit protein sequences identified.

Based on gene names obtained with UniProt, it was possible to confirm that 24 of these 66 possible extra targets for rabbits were present in our initial list of 676 rabbit X-proteins. Two of them were predicted to be rabbit proteins of interest, the glypican-3 (91.3% similarity) and the motile sperm domain-containing protein 2 (88.0% similarity). The other 22 were not selected as rabbit proteins of interest, but this opens the possibility of them being also accessible from the cell surface in rabbits. Among these 22, only 1 protein is unplaced (sodium- and chloride-dependent neutral and basic amino acid transporter B(0+), *SLC6A14*) and only 1 protein has a sequence similarity with the human protein sequence inferior to 70% (interleukin-13 receptor subunit alpha-2, 65.7% similarity). Nevertheless, the rabbit and human *IL13RA2* gene entries are considered orthologs in the NCBI database.

Since some of the 676 entries of the rabbit X chromosome proteome did not have a gene name according to UniProt, but a gene name was associated by orthology assignment using the eggNOG-mapper, the two lists were also crossed to determine if those possible extra targets matched the orthology prediction. A total of six gene names and respective protein descriptions were similar: membrane magnesium transporter 1 (*MMGT1*), moesin (*MSN*), protocadherin-11 X-linked (*PCDH11X*), cytokine receptor common subunit gamma (*IL2RG*), dystrophin (*DMD*), and ADP/ATP translocase 2 (*SLC25A5*). The proteins encoded by the orthology-assigned genes *MMGT1*, *MSN*, *PCDH11X*, and *IL2RG* were selected as possible rabbit targets and, therefore, there is a possibility that these proteins could be common with human targets instead of extra rabbit targets. Using the protein RefSeq associated with their UniProt entries, it was possible to determine that the rabbit membrane magnesium transporter 1, moesin, and protocadherin-11 X-linked shared a 99.2%, 99.3%, and 86.5% sequence similarity with the corresponding human protein sequences, respectively, and that their gene name assigned using eggNOG was the same as the one associated with their protein RefSeq. Moreover, these rabbit genes appear in the list of human orthologs from NCBI. On the other hand, the UniProt entry of the cytokine receptor common subunit gamma has no protein RefSeq associated, but the protein sequence shares 100% similarity with the rabbit entry identified by the script. The rabbit *IL2RG* associated with the RefSeq is part of the list of human orthologs of the NCBI database. Regarding the dystrophin entry, this is among the ones selected as a rabbit protein of interest. Although it has no protein RefSeq associated with the UniProt entry, the rabbit protein sequence shares 97.1% and 95.9% similarity with the rabbit and human entries for dystrophin selected by the script in NCBI, respectively. Both rabbit and human entries are part of the list of *DMD* orthologs of NCBI. Given this, there is a possibility that this protein entry (G1T8Y6) could be an extra rabbit target as predicted by the script. Regarding the protein ADP/ATP translocase 2, it was not defined as one of the rabbit proteins of interest. Its UniProt entry was also not associated with a protein RefSeq. Yet, the protein sequence shares 100% similarity with the rabbit entry and 98.1% with the human entry selected by the script. Once more, the entries selected by the script are part of the list of *SLC25A5* orthologs of NCBI. Therefore, it is possible that this protein is also accessible from the cell surface in rabbits.

In addition, 36 of the 66 proteins were not found in our initial list of rabbit X-proteins, neither using the gene name from UniProt nor the one assigned with eggNOG. Among them, six are known to be encoded by the rabbit X chromosome according to NCBI entries, with either no UniProt entry available or identified as unplaced in the UniProt database. Those six proteins are the glycoprotein Xg (*XG*), the lysophosphatidic acid receptor 4 (*LPAR4*), the kita-kyushu lung cancer antigen 1 (*CT83*), the proteolipid protein 2 (*PLP2*), the MICOS complex subunit MIC26 (*APOO*), and the synaptophysin (*SYP*). Only the kita-kyushu lung cancer antigen 1 (50.5%) and the glycoprotein Xg (47.8%) have a low sequence similarity percentage compared to the human sequence. Yet, both human and rabbit *CT83* genes and human and rabbit *XG* genes are described as orthologs in the NCBI database. The other 30 out of the 36 proteins are the ones that are still unplaced in the rabbit proteome, of which 8 proteins have a sequence similarity inferior to 70% when compared with the human sequence selected by the script. These are the anosmin-1 (*ANOS1*, 59.2%), claudin-34 (*CLDN34*, 47.7%), cytokine receptor-like factor 2 (*CRLF2*, 47.9%), granulocyte-macrophage colony-stimulating factor receptor subunit alpha (*CSF2RA*, 49.6%), interleukin-9 receptor (low-quality protein, *IL9R*, 63.0%), small integral membrane protein 9 (*SMIM9*, 69.7%), steryl-sulfatase (*STS*, 65.1%), and vesicle-associated membrane protein 7 (*VAMP7*, 66.3%). Of these, only *CLDN34* and *IL9R* rabbit genes are not described as orthologs of the respective human genes in the NCBI database. It is worth noticing that two human targets, the medium-wave-sensitive opsin 1 (*OPN1MW*) and medium-wave-sensitive opsin 2 (*OPN1MW2*), were linked by the script to the same unplaced rabbit protein, since in the NCBI database, this rabbit protein entry has as the main gene the *OPN1MW* and the *OPN1MW2* as an alias, and no individual entries were found. In humans, *OPN1MW*, *OPN1MW2*, and *OPN1MW3* are paralog genes and share 100% sequence similarity. The medium-wave-sensitive opsin 1 sequence found in the rabbit proteome shares a similarity of 87.9% with them.

As a result of this cross-species analysis, a total of 114 possible common entries were identified between humans and rabbits, of which 60 represent potential additional rabbit X-targets determined based on the human X-targets; 33 of them were encoded by the rabbit X chromosome and 27 were still unplaced in the rabbit proteome (manual curation detailed in the Supplementary Figure S2). Combining this with the previously defined list of 61 potential rabbit X-targets (Table 1) yields a comprehensive list of 121 potential rabbit X-targets. The 60 proteins added are listed below (Table 4).

### 3.4. Cross-Reference of the Common Human and Rabbit Targets with Human Spermatozoa Proteins

The list of 114 possible common human and rabbit X-targets was cross-referenced with a list of proteins described in human spermatozoa until 23 January 2023, based on a literature search previously conducted by our research group on PubMed, Scopus, and Web of Science databases [45]. Utilizing either the gene name and/or UniProt ID, 53 common proteins were identified, highlighting their potential to be used as targets of X-chromosome-bearing spermatozoa (Table 5). Among these, 24 proteins were originally identified as potential rabbit X-targets in the first analysis (Section 3.2.1, Table 1), while the remaining 29 were part of the additional possible rabbit X-targets identified based on the cross-species analysis (Section 3.3, Table 4).

## 4. Discussion

Given the significance of gaining a better understanding of the rabbit proteome and the relevance of implementing a sperm sexing technique for this species, the proteomes of the X and Y chromosomes were further explored to compile a list of proteins with the potential to be integrated into a sperm sexing technique. Although the rabbit genome OryCun 2.0, obtained from a female, was updated in 2019 with the genome assembly UM_NZW_1.0, obtained from a male and contributing to closing 75% of the gaps, only 22 chromosomes have been constructed, comprising the 21 autosomes and the X chromosome [49]. Additionally, the rabbit proteome available at UniProt contains information only for the autosomes and the X chromosome. Therefore, the relatively abundant data and annotations for proteomes of other species, such as humans, in contrast to the limited information available for the rabbit proteome, provided a valuable reference point, facilitating the interpretation and potential extrapolation of results of the rabbit. 

Effectively bridging and extrapolating information across species is a complex endeavor. It is not only important to establish a universal language for sharing biological elements but also to possess a good understanding of genetic evolution. A language that can be applied to all eukaryotes for annotation was developed by the Gene Ontology (GO) consortium. This classification system arose from the need to unify knowledge regarding the roles of genes and proteins and encompasses three key ontologies: molecular function, biological process, and cellular component [37]. The inference of structural and functional information for proteins through proteins with a common evolutionary origin (homologous) has also been widely applied through orthologous or paralogous detection [24,30,50]. While both orthologous and paralogous genes are homologous, orthologues are related by speciation and paralogues are related by duplication [24]. Although sharing orthology does not necessarily imply the conservation of gene function, in general, when the measurements are controlled, orthologues, particularly one-to-one orthologues, tend to exhibit more functional similarity than paralogues at the same level of sequence divergence (reviewed in [24,29]). Moreover, it is reported that this difference is more pronounced for the GO category ‘cellular component’ [24]. Therefore, orthologous proteins are presumed to share the same specificities, while the specificity of paralogous proteins diverges [51]. 

To enhance our comprehension of the potential cellular localization of the X and Y proteins under study, GO information was obtained from UniProt and protein annotation through orthology assignment using the eggNOG-mapper v.2.1.12. UniProt enables direct downloading of GO annotations already linked to a specific protein in a given species, either experimentally validated or inferred from electronic annotation, sequence, or structural similarity, among others. Nevertheless, few entries had GO information experimentally validated. In the present study, it was observed that none of the entries under investigation for the rabbit X chromosome and the human Y chromosome had GO annotations supported by experimental evidence in UniProt. The only exceptions were entries associated with the human X chromosome. However, out of the 740 entries for which UniProt provided GO information, only 16 were substantiated by experimental evidence. On the other hand, eggNOG is based on precomputed clusters and phylogenies inferred for each group of orthologues, enabling the annotation of large sets of sequences and minimizing the risk of transferring annotations from putative paralogous that originate from lineage-specific duplications occurring after the reference ancestral species (in-paralogs) [24,30]. Furthermore, it has been described that when compared with other tools for sequence analysis and comparison, such as BLAST and InterProScan, the eggNOG-mapper enables faster analysis, predicts a greater number of terms per protein, and yields a higher proportion of true positive (experimentally validated) assignments [52]. 

To increase the reliability of functional transfers in the present study, certain adjustments to the eggNOG-mapper default settings were made. Specifically, only annotations from one-to-one orthology relationships were considered and, regarding GO evidence, only annotations supported by experimental evidence were transferred [52]. In addition, a minimum threshold for sequence identity, query coverage, and subject coverage was set at 80%. While it is important to note that sequence similarity alone does not guarantee an evolutionary or functional relationship, some studies have suggested a tendency for a positive correlation between functional similarity and sequence similarity [42,43,44]. Specifically, it has been described that as sequence similarity exceeds a threshold of approximately 50% residue identity, the likelihood of divergent functions decreases significantly [42].

It was also part of the major aim of this study to better characterize the proteins in terms of topology since proteins present in the cell surface or transmembrane proteins present in the plasma membrane or extracellular space hold greater potential for integration into an immunological-based sperm sexing technology. For this purpose, the DeepTMHMM software was used to predict protein topology, as it is described as one of the most comprehensive and high-performing methods in comparison to similar tools [32]. The predicted transmembrane proteins were exclusively of the alpha-helical type. Notably, the rabbit and human X chromosomes exhibited similar percentages of transmembrane proteins at 19.2% and 21.2%, respectively. The results obtained for the human X chromosome proteome are closer to the results obtained in a previous study that had determined that 26% of the total human proteome were transmembrane proteins [53]. The slight discrepancy may result from the fact that we analyzed the proteome of a specific chromosome, utilized an up-to-date human reference proteome as of September 2023, as opposed to March 2013, and employed a different tool for predicting protein topology. On the other hand, only 5.3% of the human Y chromosome proteins were predicted to be transmembrane. 

Based on these results, primarily, we obtained a list of 100 rabbit X-proteins potentially associated with the plasma membrane or cell surface, as well as a list of 61 rabbit X-targets probably accessible from the cell surface. The overrepresentation analysis of the 676 entries of the rabbit X chromosome proteome indicated that the biological process ‘negative regulation of transcription by RNA polymerase II’ was overrepresented, which may be linked to the regulatory processes involved in X chromosome inactivation. It is described in the recent literature that RNA polymerase II depletion from the X chromosome is an early event during the initiation of X chromosome inactivation, aligning with a blockage of transcription [54]. Furthermore, the exclusion of RNA polymerase II from the inactive X chromosome may be attributed to chromatin modifications that can both protect or repress RNA polymerase II-binding events [54]. Membrane proteins are very important for cell function control based on their capacity to adapt to the environment. Therefore, as expected, the analysis of rabbit proteins associated with the plasma membrane and cell surface, and of those defined as possible rabbit X-targets, revealed a prevalence of biological processes associated with the transmembrane transport of ions and the regulation of biological quality and intracellular pH [55,56]. Due to the nature of these two groups, an overrepresentation of plasma membrane proteins was anticipated, as well as the underrepresentation of intracellular components [57]. Similarly, the overrepresentation of the protein class ‘G-protein coupled receptor’ and the molecular function ‘G protein-coupled peptide receptor activity’ were also expected, given that G-protein-coupled receptors are integral membrane proteins [57]. 

It was also possible to identify 211 human X-proteins and 3 human Y-proteins potentially associated with the plasma membrane or cell surface, and 132 human X-targets and 3 human Y-targets probably accessible from the cell surface. To complement the present study and possibly expand the set of potential rabbit X-targets, a cross-species analysis was performed using a script to identify potential human X-targets in the rabbit proteome. Out of the 130 characterized human targets, at least 54 proteins were found to be common to the list of rabbit X-targets and 60 proteins showed potential to also be rabbit X-targets based on the information gathered when comparing the human and rabbit entries. When looking at the potential additional targets, it is possible to observe that around 78% of them share more than 80% sequence similarity when comparing the human and rabbit protein sequences and almost half of them share more than 90% sequence similarity. This analysis resulted, therefore, in a final list of 121 potential rabbit X-targets, highlighting the benefits of this combined approach. The fact that some of these rabbit proteins were not initially selected in the first step as proteins of interest or targets, not only due to insufficient GO information but also because of undefined chromosomal location, demonstrates the possibility of these proteins being poorly annotated in the rabbit proteome. It is worth noting that, among these 60 potential additional rabbit X-targets, 27 remain unplaced in the rabbit proteome, contrary to the well-known location on the X chromosome in humans. Therefore, it is plausible but not guaranteed that they are also encoded by the X chromosome in rabbits.

Additionally, the 114 potential common human and rabbit X-targets were cross-referenced with a list of proteins already identified in human spermatozoa. This analysis revealed 53 common proteins, for which prior description in spermatozoa suggests an even more promising avenue for initial investigation of their potential as targets of X-chromosome-bearing spermatozoa. Among various studies that have attempted to identify proteins specific to X- or Y-sperm or, at least, differentially expressed [58,59,60,61,62], we found eight of these proteins described in a published patent as potential targets for sperm sexing—plasma membrane calcium-transporting ATPase 3 (*ATP2B3*), renin receptor (*ATP6AP2*), copper-transporting ATPase 1 (*ATP7A*), bombesin receptor subtype-3 (*BRS3*), Neural cell adhesion molecule L1 (*L1CAM*), membrane-associated progesterone receptor component 1 (*PGRMC1*), V-set and immunoglobulin domain-containing protein 1 (*VSIG1*), and endoplasmic reticulum membrane adapter protein XK (*XK*) [58].

Regarding the human targets obtained from the human Y chromosome proteome, as would be expected, they correspond to a significantly lower number of proteins compared to the X chromosome. In the literature, it is described that the human X chromosome has about 800 protein-coding genes, while the Y chromosome has only about 78, with the male-specific region of the Y chromosome (95% of the chromosome’s length) encoding at least 27 distinct proteins or protein families [63,64]. It is also known that several genes from the X and Y chromosomes encode similar but distinguishable proteins [63]. The proteins encoded by homologous nonrecombining genes (gametologs) found on both the X and Y chromosomes may demonstrate more than 90% sequence similarity and perform comparable functions [1]. This high similarity between protein sequences poses a challenge, as it has been reported that protein-based resources commonly contain inaccurate information, describing the expression of Y-proteins in tissues or cells that do not contain the Y chromosome, for example (reviewed in [1]). Therefore, there should be an effort toward better annotation of the databases. Moreover, antibodies targeting gametologs should be designed with these similarities in mind and validated before use, as some available antibodies may be incapable of distinguishing between the X and Y isoforms [1]. It is noteworthy that one of the possible human Y-targets (amelogenin Y isoform) was determined based on gene ontology information obtained using the eggNOG-mapper. This protein was characterized by eggNOG based on its homolog encoded by the X chromosome, the amelogenin X isoform. Nevertheless, the possibility of determining the sex of humans and pigs based on the difference in sequence between the genes *AMELY* and *AMELX* is already described in the literature [65,66]. Moreover, it is described that human *AMELY* exhibits expression levels that are only 10% of those observed for *AMELX* (as cited by [67]). The other two potential targets, Neuroligin-4 Y-linked and Protocadherin-11 Y-linked, also have an X-chromosome homolog (X-degenerated and X-transposed, respectively) and are expressed in testis [63,67].

Although our bioinformatic analysis proved beneficial in terms of determining potential targets and establishing a comparison with other species as a combined approach to gather more information, it is important to note that the quality of the annotations and information available in the databases plays a major role in the accuracy of bioinformatic studies and predictions [1,24]. Moreover, conclusions drawn from this comparison between rabbit and human proteins should be made with caution, considering the inherent biological and genetic differences between rabbits and humans. Additionally, the data we utilized for the main bioinformatic analysis were sourced from several cell types rather than spermatozoa-specific datasets. Since spermatozoa are a distinct cell type with specialized functions and protein expression patterns that differentiate them from somatic cells, the functional significance of proteins within spermatozoa may diverge. Therefore, even if using good functional annotation tools and refined settings, further experimental validation is necessary to confirm the potential usefulness of these proteins to sex rabbit sperm samples.

## 5. Conclusions

This study provides valuable insights into the proteomic profiles of rabbit X-proteins, as well as human X- and Y-proteins, culminating in the obtention of a list of proteins potentially accessible from the cell surface that can possibly be used as sex-specific targets. Moreover, it demonstrated the advantages of cross-referencing the information available for other species. This cross-referencing not only enabled the determination of a list of 61 potential rabbit X-targets based on known information for rabbit proteins from UniProt and fast orthology assignments but it also allowed for the obtention of a list of 60 other rabbit proteins that may possess the characteristics necessary to be X-targets, identified through their similarity to the human entries. A complementary analysis also revealed that at least 53 potential common human and rabbit X-targets were already identified in human spermatozoa, emphasizing their potential for use as targets of X-chromosome-bearing spermatozoa. 

Future work should prioritize collecting proteomic data from rabbit spermatozoa for a more precise and comprehensive understanding of these proteins in the context of sperm biology. This will open the possibility of exploring the use of some of these proteins for rabbit sperm sexing applications.

## Figures and Tables

**Table 1:** List of the 61 proteins associated with the rabbit X chromosome that have been identified as potentially accessible from the cell surface through bioinformatic analysis. TM, alpha-helical transmembrane protein; SP, signal peptide; SP+TM, alpha-helical transmembrane protein with signal peptide; GLOB, globular protein; GO terms, Gene Ontology terms related to the plasma membrane, cell surface, or extracellular space; PM, plasma membrane; CS, cell surface; ESPM, external side of plasma membrane; ACPM, anchored component of plasma membrane; ACESPM, anchored component of external side of plasma membrane; ICPM, integral component of plasma membrane; ES, extracellular space; * preferred name annotated with eggNOG-mapper v.2.1.12 tool (one-to-one orthology, e-value 3.83 × 10^−41^ to 0.0), as no gene name was available in UniProt; ^†^ selected based on GO with experimental evidence inferred using eggNOG.

Protein ID	Gene Name	Protein Name	Topology	GO Terms
G1TEF4	*ACE2*	Angiotensin-converting enzyme	SP+TM	CS, PM, ES ^†^
G1SSU5	*ADGRG2*	Adhesion G protein-coupled receptor G2	SP+TM	PM ^†^
Q8MKE9	*AGTR2*	Type-2 angiotensin II receptor	TM	PM ^†^
G1SMQ1	*AMOT*	Angiomotin	GLOB	CS, PM, ESPM ^†^
G1T923	*ATP6AP2*	Renin receptor	SP+TM	CS, PM, ESPM ^†^
G1T6U3	*ATP7A*	P-type Cu(+) transporter	TM	PM ^†^
G1TUV0	*BRS3*	Bombesin receptor subtype-3	TM	PM ^†^
G1SNI0	*CACNA1F*	Calcium voltage-gated channel subunit alpha1 F	TM	PM ^†^
G1SV48	CD24 *	CD24 molecule	SP	CS, PM, ESPM, ACPM, ACESPM ^†^
G1SKP7	*CD40LG*	CD40 ligand	TM	CS, PM, ESPM, ES ^†^
Q9TTU3	*CLCN5*	H(+)/Cl(−) exchange transporter 5	TM	PM ^†^
G1TSU9	*CLDN2*	Claudin 2	TM	PM
G1T4N6	*CLTRN*	Collectrin, amino acid transport regulator	SP+TM	PM ^†^
A0A5F9CEX7	*CXCR3*	C-X-C chemokine receptor type 3	TM	CS, PM, ESPM ^†^
A0A5F9CNI6	*CYBB*	Cytochrome b-245 beta chain	TM	PM, ICPM ^†^
G1TB31	*CYSLTR1*	Cysteinyl leukotriene receptor 1	TM	PM, ICPM ^†^
G1T9S6	*EDA*	Ectodysplasin A	TM	PM, ES, ICPM ^†^
A0A5F9D0L9	*EDA2R*	Ectodysplasin A2 receptor	SP+TM	PM
A0A5F9CVB5	*EFNB1*	Ephrin B1	SP+TM	PM^†^
G1TPG8	*ENOX2*	Ecto-NOX disulfide-thiol exchanger 2	GLOB	CS, PM, ESPM ^†^
A0A5F9CPD1	*GDPD2*	Glycerophosphodiester phosphodiesterase domain containing 2	TM	PM ^†^
G1TMB8	*GJB1*	Gap junction protein	TM	PM ^†^
G1T1J4	*GLRA2*	Glycine receptor alpha 2	SP+TM	PM, ICPM ^†^
G1T836	GLRA4 *	Glycine receptor alpha 4	SP+TM	PM, ICPM ^†^
A0A5F9DIT5	*GPC4*	Glypican 4	TM	CS, PM, ESPM ^†^
G1SN36	*GPR174*	G protein-coupled receptor 174	TM	PM
G1SD16	*GRIA3*	Glutamate receptor	SP+TM	PM, ICPM ^†^
G1T4A8	*GRPR*	Gastrin-releasing peptide receptor	TM	PM, ICPM ^†^
G1SXP0	*GUCY2F*	Guanylate cyclase	SP+TM	PM ^†^
A0A5F9C5Z9	*HEPH*	Hephaestin	SP+TM	PM ^†^
A0A5F9DJ94	HNRNPM *	RRM domain-containing protein	GLOB	CS^†^
G1TAG7	*HTR2C*	5-hydroxytryptamine receptor 2C	SP+TM	CS, PM, ESPM, ICPM ^†^
G1TBS6	*IL1RAPL1*	Interleukin-1 receptor accessory protein-like 1	SP+TM	CS, PM ^†^
G1TPE7	*IL2RG* *	Cytokine receptor common subunit gamma	TM	CS, PM, ESPM ^†^
G1SVL1	*ITM2A*	Integral membrane protein 2	TM	PM
G1TSW5	*KCNE5*	Potassium voltage-gated channel subfamily E regulatory subunit 5	TM	PM, ICPM ^†^
U3KP42	MMGT1 *	Membrane magnesium transporter	TM	PM ^†^
G1SCP8	MSN *	Moesin	GLOB	CS, PM ^†^
G1TU56	*NDP*	Norrin cystine knot growth factor NDP	SP	CS, ES ^†^
G1TYL9	*NLGN3*	Neuroligin 3	SP+TM	CS, PM, ICPM ^†^
G1SPD7	*NOX1*	NADPH oxidase 1	TM	PM, ICPM ^†^
G1T124	*OR13H1*	Olfactory receptor family 13 subfamily H member 1	TM	PM
G1TCY0	*P2RY4*	P2Y purinoceptor 4	TM	PM ^†^
A0A5F9DQS9	PCDH11X *	Cadherin domain-containing protein	SP+TM	PM
G1TAD7	*PCDH19*	Protocadherin 19	SP+TM	PM
G1SQ59	*PHEX*	Phosphate-regulating endopeptidase homolog X-linked	TM	CS ^†^
G1T949	*PORCN*	Porcupine O-acyltransferase	TM	PM, ICPM ^†^
G1T7A1	*PTCHD1*	Patched domain-containing 1	TM	PM ^†^
G1SZZ7	*RS1*	Retinoschisin 1	SP	PM, ESPM, ES
G1SZJ1	*SLC7A3*	Solute carrier family 7 member 3	TM	PM ^†^
G1T4W3	*SLC9A6*	Sodium/hydrogen exchanger	SP+TM	PM ^†^
G1SLC9	*SLC9A7*	Sodium/hydrogen exchanger	SP+TM	PM
G1T0T4	*SRPX2*	Sushi repeat containing protein X-linked 2	SP	CS, PM, ES ^†^
G1SN13	*TENM1*	Teneurin transmembrane protein 1	TM	PM, ICPM ^†^
O62852	*TRPC5*	Short transient receptor potential channel 5	TM	PM
A0A5F9CK03	*VSIG1*	V-set and immunoglobulin domain-containing protein 1	SP+TM	PM ^†^
G1SR92	*XK*	XK-related protein	TM	PM
A0A5F9CW02	-	Transmembrane protein 182	TM	PM
G1THX1	-	Sodium/hydrogen exchanger	TM	PM
G1TL60	-	Olfactory receptor	TM	PM
U3KP07	-	receptor protein-tyrosine kinase	TM	PM

**Table 2:** List of the 132 proteins associated with the human X chromosome that have been identified as potentially accessible from the cell surface through bioinformatic analysis. TM, alpha-helical transmembrane protein; SP, signal peptide; SP+TM, alpha-helical transmembrane protein with signal peptide; GLOB, globular protein; GO terms, Gene Ontology terms related to the plasma membrane, cell surface, or extracellular space; PM, plasma membrane; CS, cell surface; ESPM, external side of plasma membrane; ACPM, anchored component of plasma membrane; ICPM, integral component of plasma membrane; ES, extracellular space; * preferred name annotated with eggNOG-mapper v.2.1.12 tool (one-to-one orthology, e-value 2.11 × 10^−232^ and 1.55 × 10^−59^), as no gene name was available in UniProt; ^†^ selected based on GO with experimental evidence inferred using eggNOG; ^††^ selected based on GO with experimental evidence obtained through UniProt and inferred using eggNOG.

Protein ID	Gene Name	Protein Name	Topology	GO Terms
Q9BYF1	*ACE2*	Angiotensin-converting enzyme 2	SP+TM	CS, PM, ES ^††^
Q8IZP9	*ADGRG2*	Adhesion G-protein-coupled receptor G2	SP+TM	CS, PM ^†^
P50052	*AGTR2*	Type-2 angiotensin II receptor	TM	PM ^†^
Q99217	*AMELX*	Amelogenin, X isoform	SP	CS ^†^
Q4VCS5	*AMOT*	Angiomotin	GLOB	CS, PM, ESPM ^†^
P23352	*ANOS1*	Anosmin-1	SP	CS, PM, ESPM, ES ^†^
Q9BUR5	*APOO*	MICOS complex subunit MIC26	TM	ES
Q8NB49	*ATP11C*	Phospholipid-transporting ATPase IG	TM	PM ^†^
Q9UN42	*ATP1B4*	Protein ATP1B4	TM	PM
Q16720	*ATP2B3*	Plasma membrane calcium-transporting ATPase 3	TM	PM ^†^
Q15904	*ATP6AP1*	V-type proton ATPase subunit S1	SP+TM	PM ^†^
O75787	*ATP6AP2*	Renin receptor	SP+TM	CS, PM, ESPM ^†^
Q04656	*ATP7A*	Copper-transporting ATPase 1	TM	PM^†^
P30518	*AVPR2*	Vasopressin V2 receptor	TM	PM
P51572	*BCAP31*	B-cell receptor-associated protein 31	TM	PM, ICPM ^†^
P21810	*BGN*	Biglycan	SP	CS, PM, ES ^†^
P32247	*BRS3*	Bombesin receptor subtype-3	TM	PM ^†^
O60840	*CACNA1F*	Voltage-dependent L-type calcium channel subunit alpha-1F	TM	PM ^†^
P29965	*CD40LG*	CD40 ligand	TM	CS, PM, ESPM, ES ^†^
P14209	*CD99*	CD99 antigen	SP+TM	PM
Q8TCZ2	*CD99L2*	CD99 antigen-like protein 2	SP+TM	CS, PM ^†^
P51795	*CLCN5*	H(+)/Cl(−) exchange transporter 5	TM	PM ^†^
P57739	*CLDN2*	Claudin-2	TM	PM
H7C241	*CLDN34*	Claudin-34	TM	PM
Q9HBJ8	*CLTRN*	Collectrin	SP+TM	PM ^†^
A0A3B3IT09	*CLTRN* *	Collectrin domain-containing protein	TM	PM
Q16280	*CNGA2*	Cyclic nucleotide-gated olfactory channel	TM	PM
Q9HC73	*CRLF2*	Cytokine receptor-like factor 2	SP+TM	CS, PM, ESPM ^†^
P15509	*CSF2RA*	Granulocyte-macrophage colony-stimulating factor receptor subunit alpha	SP+TM	PM, ESPM ^†^
Q5H943	*CT83*	Kita-kyushu lung cancer antigen 1	TM	PM
P49682	*CXCR3*	C-X-C chemokine receptor type 3	TM	CS, PM, ESPM ^†^
P04839	*CYBB*	Cytochrome b-245 heavy chain	TM	PM, ICPM ^†^
Q9Y271	*CYSLTR1*	Cysteinyl leukotriene receptor 1	TM	PM, ICPM ^†^
P11532	*DMD*	Dystrophin	GLOB	CS, PM ^†^
Q92838	*EDA*	Ectodysplasin-A	TM	PM, ES, ICPM ^†^
Q9HAV5	*EDA2R*	Tumor necrosis factor receptor superfamily member 27	TM	PM
P98172	*EFNB1*	Ephrin-B1	SP+TM	CS, PM ^†^
Q16206	*ENOX2*	Ecto-NOX disulfide-thiol exchanger 2	GLOB	CS, PM, ESPM, ES ^†^
P34903	*GABRA3*	Gamma-aminobutyric acid receptor subunit alpha-3	SP+TM	PM
Q9UN88	*GABRQ*	Gamma-aminobutyric acid receptor subunit theta	SP+TM	PM
Q9HCC8	*GDPD2*	Glycerophosphoinositol inositolphosphodiesterase	TM	PM ^†^
P08034	*GJB1*	Gap junction beta-1 protein	TM	PM ^†^
P23416	*GLRA2*	Glycine receptor subunit alpha-2	SP+TM	PM, ICPM ^†^
P51654	*GPC3*	Glypican-3	SP	CS, PM, ACPM
O75487	*GPC4*	Glypican-4	SP	CS, PM, ESPM ^†^
Q13491	*GPM6B*	Neuronal membrane glycoprotein M6-b	TM	PM ^†^
Q96P66	*GPR101*	Probable G-protein-coupled receptor 101	TM	PM
Q8TDV5	*GPR119*	Glucose-dependent insulinotropic receptor	TM	PM
P51810	*GPR143*	G-protein-coupled receptor 143	TM	PM ^†^
Q9NS66	*GPR173*	Probable G-protein-coupled receptor 173	TM	PM
Q9BXC1	*GPR174*	Probable G-protein-coupled receptor 174	TM	PM
Q9UPC5	*GPR34*	Probable G-protein-coupled receptor 34	TM	PM
Q13585	*GPR50*	Melatonin-related receptor	TM	PM ^†^
Q96P67	*GPR82*	Probable G-protein-coupled receptor 82	TM	PM
P42263	*GRIA3*	Glutamate receptor 3	SP+TM	PM, ICPM ^†^
P30550	*GRPR*	Gastrin-releasing peptide receptor	TM	PM, ICPM ^†^
P51841	*GUCY2F*	Retinal guanylyl cyclase 2	SP+TM	PM ^†^
Q9BQS7	*HEPH*	Hephaestin	SP+TM	PM ^†^
P28335	*HTR2C*	5-hydroxytryptamine receptor 2C	SP+TM	CS, PM, ESPM, ICPM ^†^
P78552	*IL13RA1*	Interleukin-13 receptor subunit alpha-1	SP+TM	PM, ESPM
Q14627	*IL13RA2*	Interleukin-13 receptor subunit alpha-2	SP+TM	ESPM, ES
Q9NZN1	*IL1RAPL1*	Interleukin-1 receptor accessory protein-like 1	SP+TM	CS, PM ^†^
Q9NP60	*IL1RAPL2*	X-linked interleukin-1 receptor accessory protein-like 2	SP+TM	PM
P31785	*IL2RG*	Cytokine receptor common subunit gamma	SP+TM	CS, PM, ESPM ^†^
A0A2R8YE73	*IL2RG* *	Fibronectin type-III domain-containing protein	SP+TM	CS, PM, ESPM ^†^
P26951	*IL3RA*	Interleukin-3 receptor subunit alpha	SP+TM	PM, ESPM
Q01113	*IL9R*	Interleukin-9 receptor	SP+TM	PM, ESPM, ES
P51617	*IRAK1*	Interleukin-1 receptor-associated kinase 1	GLOB	CS, PM
O43736	*ITM2A*	Integral membrane protein 2A	TM	PM
Q9NSA2	*KCND1*	Potassium voltage-gated channel subfamily D member 1	TM	PM
Q9UJ90	*KCNE5*	Potassium voltage-gated channel subfamily E regulatory beta subunit 5	TM	PM, ICPM ^†^
P32004	*L1CAM*	Neural cell adhesion molecule L1	SP+TM	CS, PM, ESPM ^†^
P13473	*LAMP2*	Lysosome-associated membrane glycoprotein 2	SP+TM	PM, ES ^†^
Q99677	*LPAR4*	Lysophosphatidic acid receptor 4	TM	PM ^†^
Q9H0U3	*MAGT1*	Magnesium transporter protein 1	SP+TM	PM
Q8N4V1	*MMGT1*	ER membrane protein complex subunit 5	TM	PM ^†^
Q8NHP6	*MOSPD2*	Motile sperm domain-containing protein 2	TM	PM, ICPM ^†^
P26038	*MSN*	Moesin	GLOB	CS, PM, ES ^†^
O75949	*NALF2*	NALCN channel auxiliary factor 2	TM	PM
Q00604	*NDP*	Norrin	SP	CS, ES ^†^
Q9NZ94	*NLGN3*	Neuroligin-3	SP+TM	CS, PM, ICPM ^†^
Q8N0W4	*NLGN4X*	Neuroligin-4, X-linked	SP+TM	CS, PM, ICPM ^†^
Q9Y5S8	*NOX1*	NADPH oxidase 1	TM	PM, ICPM ^†^
P04000	*OPN1LW*	Long-wave-sensitive opsin 1	TM	PM
P04001	*OPN1MW*	Medium-wave-sensitive opsin 1	TM	PM
P0DN77	*OPN1MW2*	Medium-wave-sensitive opsin 2	TM	PM
P0DN78	*OPN1MW3*	Medium-wave-sensitive opsin 3	TM	PM
Q8NG92	*OR13H1*	Olfactory receptor 13H1	TM	PM
O00398	*P2RY10*	Putative P2Y purinoceptor 10	TM	PM
P51582	*P2RY4*	P2Y purinoceptor 4	TM	PM^†^
Q86VZ1	*P2RY8*	P2Y purinoceptor 8	TM	PM
Q9BZA7	*PCDH11X*	Protocadherin-11 X-linked	SP+TM	PM
Q8TAB3	*PCDH19*	Protocadherin-19	SP+TM	PM
O00264	*PGRMC1*	Membrane-associated progesterone receptor component 1	TM	PM
P78562	*PHEX*	Phosphate-regulating neutral endopeptidase PHEX	TM	CS, PM ^†^
P60201	*PLP1*	Myelin proteolipid protein	TM	PM ^†^
Q04941	*PLP2*	Proteolipid protein 2	TM	PM ^†^
P51805	*PLXNA3*	Plexin-A3	SP+TM	PM ^†^
Q9ULL4	*PLXNB3*	Plexin-B3	SP+TM	CS, PM ^†^
Q9H237	*PORCN*	Protein-serine O-palmitoleoyltransferase porcupine	TM	PM, ICPM ^†^
O14668	*PRRG1*	Transmembrane gamma-carboxyglutamic acid protein 1	TM	PM, ES
Q9BZD7	*PRRG3*	Transmembrane gamma-carboxyglutamic acid protein 3	TM	ES
Q96NR3	*PTCHD1*	Patched domain-containing protein 1	TM	PM ^†^
O15537	*RS1*	Retinoschisin	SP	PM, ESPM, ES
P36021	*SLC16A2*	Monocarboxylate transporter 8	TM	PM, ICPM ^†^
O95258	*SLC25A14*	Brain mitochondrial carrier protein 1	TM	PM
P05141	*SLC25A5*	ADP/ATP translocase 2	TM	PM
Q8WUX1	*SLC38A5*	Sodium-coupled neutral amino acid transporter 5	TM	PM
Q9UN76	*SLC6A14*	Sodium- and chloride-dependent neutral and basic amino acid transporter B(0+)	TM	PM
P48029	*SLC6A8*	Sodium- and chloride-dependent creatine transporter 1	TM	PM
Q8WY07	*SLC7A3*	Cationic amino acid transporter 3	TM	PM ^†^
Q92581	*SLC9A6*	Sodium/hydrogen exchanger 6	SP+TM	PM ^†^
Q96T83	*SLC9A7*	Sodium/hydrogen exchanger 7	SP+TM	PM
Q9H156	*SLITRK2*	SLIT- and NTRK-like protein 2	SP+TM	PM
Q8IW52	*SLITRK4*	SLIT- and NTRK-like protein 4	SP+TM	PM
A6NGZ8	*SMIM9*	Small integral membrane protein 9	SP+TM	PM
P78539	*SRPX*	Sushi repeat-containing protein SRPX	SP	CS
O60687	*SRPX2*	Sushi repeat-containing protein SRPX2	SP	CS, PM, ES ^†^
P08842	*STS*	Steryl-sulfatase	SP+TM	PM
P08247	*SYP*	Synaptophysin	TM	PM ^†^
P51864	*TDGF1P3*	Putative teratocarcinoma-derived growth factor 3	SP	CS, PM, ES ^†^
Q9UKZ4	*TENM1*	Teneurin-1	TM	PM, ICPM ^†^
Q9NYK1	*TLR7*	Toll-like receptor 7	SP+TM	PM ^†^
Q9NR97	*TLR8*	Toll-like receptor 8	SP+TM	CS, PM, ESPM ^†^
Q9BQJ4	*TMEM47*	Transmembrane protein 47	TM	PM ^†^
Q9UL62	*TRPC5*	Short transient receptor potential channel 5	TM	PM
P41732	*TSPAN7*	Tetraspanin-7	TM	PM
P51809	*VAMP7*	Vesicle-associated membrane protein 7	TM	CS, PM ^†^
Q86XK7	*VSIG1*	V-set and immunoglobulin domain-containing protein 1	SP+TM	PM ^†^
P55808	*XG*	Glycoprotein Xg	SP+TM	PM, ICPM ^†^
P51811	*XK*	Endoplasmic reticulum membrane adapter protein XK	TM	PM
Q6PP77	*XKRX*	XK-related protein 2	TM	PM

**Table 3:** List of the 3 proteins associated with the human Y chromosome that have been identified as potentially accessible from the cell surface through bioinformatic analysis. SP, signal peptide; SP+TM, alpha-helical transmembrane protein with signal peptide; GO terms, Gene Ontology terms related to the plasma membrane, cell surface, or extracellular space; PM, plasma membrane; CS, cell surface, ^†^ selected based on GO with experimental evidence inferred using eggNOG.

Protein ID	Gene Name	Protein Name	Topology	GO Terms
Q99218	*AMELY*	Amelogenin, Y isoform	SP	CS ^†^
Q8NFZ3	*NLGN4Y*	Neuroligin-4, Y-linked	SP+TM	CS, PM
Q9BZA8	*PCDH11Y*	Protocadherin-11, Y-linked	SP+TM	PM

**Table 4:** List of the 60 potential additional rabbit targets of the X chromosome determined based on the human targets of the X chromosome. For each possible additional target, the protein reference sequence identifier of the NCBI database (RefSeq) for the human and rabbit entries, gene name, the protein description, protein sequence similarity between humans and rabbits (%), and the associated rabbit chromosome are described.

RefSeq_Human	RefSeq_Rabbit	Gene Name	Protein Description	Similarity (%)	Chromosome
XP_054183011	XP_051689653	*ANOS1*	anosmin-1	59.2	Unplaced
XP_054183834	XP_051683595	*APOO*	MICOS complex subunit MIC26	85.4	X
XP_054182860	XP_051687539	*ATP11C*	phospholipid-transporting ATPase IG	97.3	Unplaced
XP_016884870	XP_008271374	*ATP1B4*	protein ATP1B4	87.0	X
XP_016885042	XP_051693419	*ATP2B3*	plasma membrane calcium-transporting ATPase 3	90.5	Unplaced
NP_001174	NP_001164848	*ATP6AP1*	V-type proton ATPase subunit S1 precursor	90.4	Unplaced
NP_000045	XP_008248538	*AVPR2*	vasopressin V2 receptor	86.0	Unplaced
NP_001132929	XP_008248542	*BCAP31*	B-cell receptor-associated protein 31	92.7	Unplaced
XP_054183538	XP_051693405	*BGN*	biglycan	94.0	Unplaced
XP_047298518	XP_008273252	*CD99L2*	CD99 antigen-like protein 2	76.5	Unplaced
NP_005131	XP_051689801	*CNGA2*	cyclic nucleotide-gated olfactory channel	94.0	Unplaced
XP_011544483	XP_008273625	*CRLF2*	cytokine receptor-like factor 2	47.9	Unplaced
XP_047297804	XP_008249542	*CSF2RA*	granulocyte-macrophage colony-stimulating factor receptor subunit alpha	49.6	Unplaced
NP_001017978	XP_008271135	*CT83*	kita-kyushu lung cancer antigen 1	50.5	X
XP_006724531	XP_051683632	*DMD*	dystrophin	94.6	X
XP_054182733	XP_051689815	*GABRA3*	gamma-aminobutyric acid receptor subunit alpha-3	89.7	Unplaced
XP_011529486	XP_002721381	*GABRQ*	gamma-aminobutyric acid receptor subunit theta	84.7	Unplaced
XP_054182809	XP_002720349	*GPC3*	glypican-3	91.3	X
NP_473362	XP_017205202	*GPR101*	probable G-protein-coupled receptor 101	84.8	X
NP_848566	XP_002720339	*GPR119*	glucose-dependent insulinotropic receptor	85.1	X
XP_054183230	XP_008270867	*GPR173*	probable G-protein-coupled receptor 173	100.0	X
XP_005272654	XP_002719905	*GPR34*	probable G-protein-coupled receptor 34	90.9	X
XP_011529518	XP_008273247	*GPR50*	melatonin-related receptor	74.7	Unplaced
XP_047297947	XP_051682990	*GPR82*	probable G-protein-coupled receptor 82	90.8	X
XP_054183006	XP_008271141	*IL13RA1*	interleukin-13 receptor subunit alpha-1	83.8	X
XP_054183007	XP_051683230	*IL13RA2*	interleukin-13 receptor subunit alpha-2	65.7	X
XP_011529207	XP_008271052	*IL1RAPL2*	X-linked interleukin-1 receptor accessory protein-like 2	95.4	X
XP_047298053	XP_051693453	*IRAK1*	interleukin-1 receptor-associated kinase 1	82.7	Unplaced
NP_004970	XP_002719951	*KCND1*	potassium voltage-gated channel subfamily D member 1	97.1	X
NP_000416	XP_051693407	*L1CAM*	neural cell adhesion molecule L1	91.3	Unplaced
NP_001116078	XP_008271376	*LAMP2*	lysosome-associated membrane glycoprotein 2	84.2	X
XP_016884927	XP_008271183	*LPAR4*	lysophosphatidic acid receptor 4	98.4	X
NP_001354845	XP_008271178	*MAGT1*	magnesium transporter protein 1	97.3	X
NP_689794	XP_051683552	*MOSPD2*	motile sperm domain-containing protein 2	88.0	X
XP_054182806	XP_051683121	*NALF2*	NALCN channel auxiliary factor 2	86.7	X
NP_000504	NP_001309193	*OPN1MW*	medium-wave-sensitive opsin 1	87.9	Unplaced
XP_005274486	XP_002724295	*P2RY8*	P2Y purinoceptor 8	78.9	Unplaced
XP_047297954	XP_008271184	*P2RY10*	putative P2Y receptor family member 10	87.9	X
NP_006658	XP_002720306	*PGRMC1*	membrane-associated progesterone receptor component 1	93.8	X
NP_000524	XP_008271259	*PLP1*	myelin proteolipid protein	99.6	X
NP_002659	NP_001075566	*PLP2*	proteolipid protein 2	88.2	X
XP_047298203	XP_008248506	*PLXNA3*	plexin-A3	88.5	Unplaced
NP_005384	XP_017194059	*PLXNB3*	plexin-B3	89.0	Unplaced
NP_001135867	XP_008270716	*PRRG1*	transmembrane gamma-carboxyglutamic acid protein 1	96.3	X
NP_006508	XP_051691041	*SLC16A2*	monocarboxylate transporter 8	96.8	Unplaced
XP_011529704	XP_008271409	*SLC25A14*	brain mitochondrial carrier protein 1	91.6	X
NP_001143	XP_002720308	*SLC25A5*	ADP/ATP translocase 2	98.3	X
XP_054184094	XP_051682846	*SLC38A5*	sodium-coupled neutral amino acid transporter 5	83.9	X
NP_009162	XP_002720288	*SLC6A14*	sodium- and chloride-dependent neutral and basic amino acid transporter B(0+)	90.8	X
NP_005620	NP_001075866	*SLC6A8*	sodium- and chloride-dependent creatine transporter 1	97.5	Unplaced
XP_047298533	XP_051687602	*SLITRK2*	SLIT and NTRK-like protein 2	98.3	Unplaced
XP_054182427	XP_008271667	*SLITRK4*	SLIT and NTRK-like protein 4	98.1	Unplaced
NP_001156408	XP_051691991	*SMIM9*	small integral membrane protein 9	69.7	Unplaced
XP_016885382	XP_051682971	*SRPX*	sushi repeat-containing protein SRPX, partial	94.6	X
XP_047298063	XP_008247483	*STS*	steryl-sulfatase	65.1	Unplaced
NP_003170	XP_051682874	*SYP*	synaptophysin	79.8	X
NP_004606	XP_017205349	*TSPAN7*	tetraspanin-7	99.2	X
XP_011529490	XP_002722247	*VAMP7*	vesicle-associated membrane protein 7	66.3	Unplaced
XP_005274644	XP_008271435	*XG*	glycoprotein Xg	47.8	X
XP_011529256	XP_002720425	*XKRX*	XK-related protein 2	91.2	X

**Table 5:** List of the 53 common human and rabbit potential targets of the X chromosome previously described in human spermatozoa. For each target, the UniProt entry ID (*Homo sapiens*), gene name, and protein name are described. * Target with gene encoded by the X chromosome in rabbits, ^†^ target present in the first list of rabbit X-targets obtained (Table 1).

Entry	Gene Name	Protein Name
Q9BYF1	*ACE2* *^†^	Angiotensin-converting enzyme 2
Q8IZP9	*ADGRG2* *^†^	Adhesion G-protein-coupled receptor G2
Q4VCS5	*AMOT* *^†^	Angiomotin
P23352	*ANOS1*	Anosmin-1
Q9BUR5	*APOO* *	MICOS complex subunit MIC26
Q8NB49	*ATP11C*	Phospholipid-transporting ATPase IG
Q16720	*ATP2B3*	Plasma membrane calcium-transporting ATPase 3
Q15904	*ATP6AP1*	V-type proton ATPase subunit S1
O75787	*ATP6AP2* *^†^	Renin receptor
Q04656	*ATP7A* *^†^	Copper-transporting ATPase 1
P51572	*BCAP31*	B-cell receptor-associated protein 31
P21810	*BGN*	Biglycan
P32247	*BRS3* *^†^	Bombesin receptor subtype-3
O60840	*CACNA1F* *^†^	Voltage-dependent L-type calcium channel subunit alpha-1F
P51795	*CLCN5* *^†^	H(+)/Cl(−) exchange transporter 5
P57739	*CLDN2* *^†^	Claudin-2
Q9HC73	*CRLF2*	Cytokine receptor-like factor 2
Q5H943	*CT83* *	Kita-kyushu lung cancer antigen 1
P04839	*CYBB* *^†^	Cytochrome b-245 heavy chain
P11532	*DMD* *	Dystrophin
Q92838	*EDA* *^†^	Ectodysplasin-A
Q16206	*ENOX2* *^†^	Ecto-NOX disulfide-thiol exchanger 2
P34903	*GABRA3*	Gamma-aminobutyric acid receptor subunit alpha-3
Q9HCC8	*GDPD2* *^†^	Glycerophosphoinositol inositolphosphodiesterase GDPD2
P51654	*GPC3* *	Glypican-3
O75487	*GPC4* *^†^	Glypican-4
Q14627	*IL13RA2* *	Interleukin-13 receptor subunit alpha-2
Q9NZN1	*IL1RAPL1* *^†^	Interleukin-1 receptor accessory protein-like 1
P51617	*IRAK1*	Interleukin-1 receptor-associated kinase 1
P32004	*L1CAM*	Neural cell adhesion molecule L1
P13473	*LAMP2* *	Lysosome-associated membrane glycoprotein 2
Q9H0U3	*MAGT1* *	Magnesium transporter protein 1
Q8N4V1	*MMGT1* *^†^	ER membrane protein complex subunit 5
Q8NHP6	*MOSPD2* *	Motile sperm domain-containing protein 2
P26038	*MSN* *^†^	Moesin
Q00604	*NDP* *^†^	Norrin
Q9Y5S8	*NOX1* *^†^	NADPH oxidase 1
O00264	*PGRMC1* *	Membrane-associated progesterone receptor component 1
Q04941	*PLP2* *	Proteolipid protein 2
Q96NR3	*PTCHD1* *^†^	Patched domain-containing protein 1
O95258	*SLC25A14* *	Brain mitochondrial carrier protein 1
P05141	*SLC25A5* *	ADP/ATP translocase 2
Q9UN76	*SLC6A14* *	Sodium- and chloride-dependent neutral and basic amino acid transporter B(0+)
Q92581	*SLC9A6* *^†^	Sodium/hydrogen exchanger 6
Q9H156	*SLITRK2*	SLIT and NTRK-like protein 2
P78539	*SRPX* *	Sushi repeat-containing protein SRPX
P08842	*STS*	Steryl-sulfatase
P08247	*SYP* *	Synaptophysin
Q9UKZ4	*TENM1* *^†^	Teneurin-1
P41732	*TSPAN7* *	Tetraspanin-7
P51809	*VAMP7*	Vesicle-associated membrane protein 7
Q86XK7	*VSIG1* *^†^	V-set and immunoglobulin domain-containing protein 1
P51811	*XK* *^†^	Endoplasmic reticulum membrane adapter protein XK

## Data Availability

The data presented in this study are available in article and Supplementary Material.

## Supplementary Materials

The following supporting information can be downloaded at: https://www.mdpi.com/article/10.3390/ani14020217/s1, Figure S1: Representative scheme of the number of proteins of interest obtained for the rabbit X chromosome and human X and Y chromosomes, based on Gene Ontology information obtained through UniProt and orthology assignments using eggNOG v.2.1.12. Additionally, it indicates the number of proteins that may be accessible from the cell surface (potential targets) after incorporating the results of protein topology prediction from the DeepTMHMM v.1.0.24 software; Figure S2: Schematization of the manual curation of the data obtained using the script designed for the cross-species analysis to identify human targets in the rabbit proteome; Spreadsheet S1: Characterization of the proteins of interest and possible targets (green) of the rabbit X chromosome proteome and human X and Y chromosomes proteome.
