# Supplementary figures and images for: Comparative Bioinformatic Analysis of the Proteomes of Rabbit and Human Sex Chromosomes
Source: Animals (Basel). 2024 Jan 9;14(2):217. doi: 10.3390/ani14020217 (PMC10812427; doi:10.3390/ani14020217)

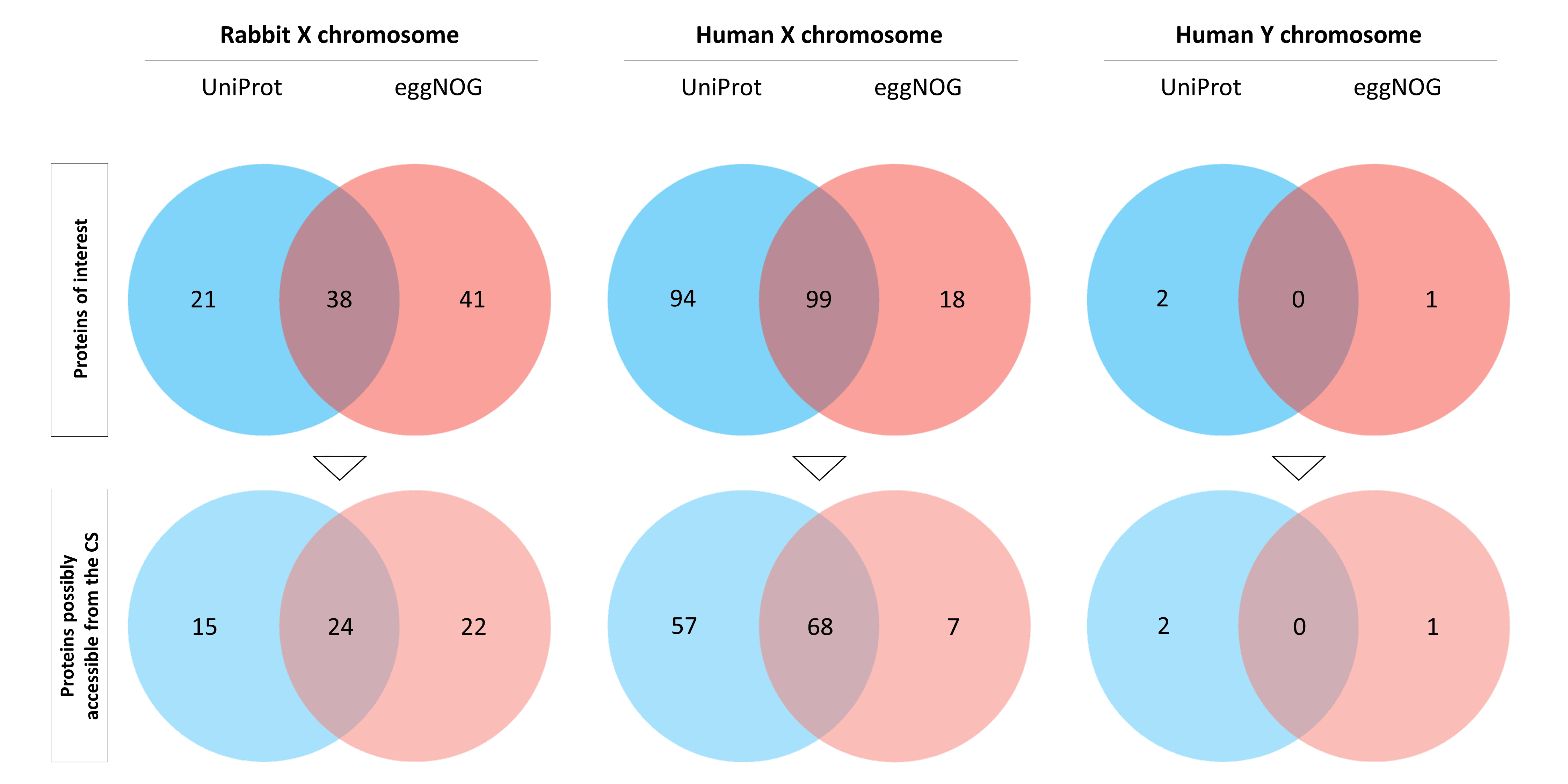

Supplement: Supplementary file 1 [file animals-14-00217-s001.zip › Figure S1.png]

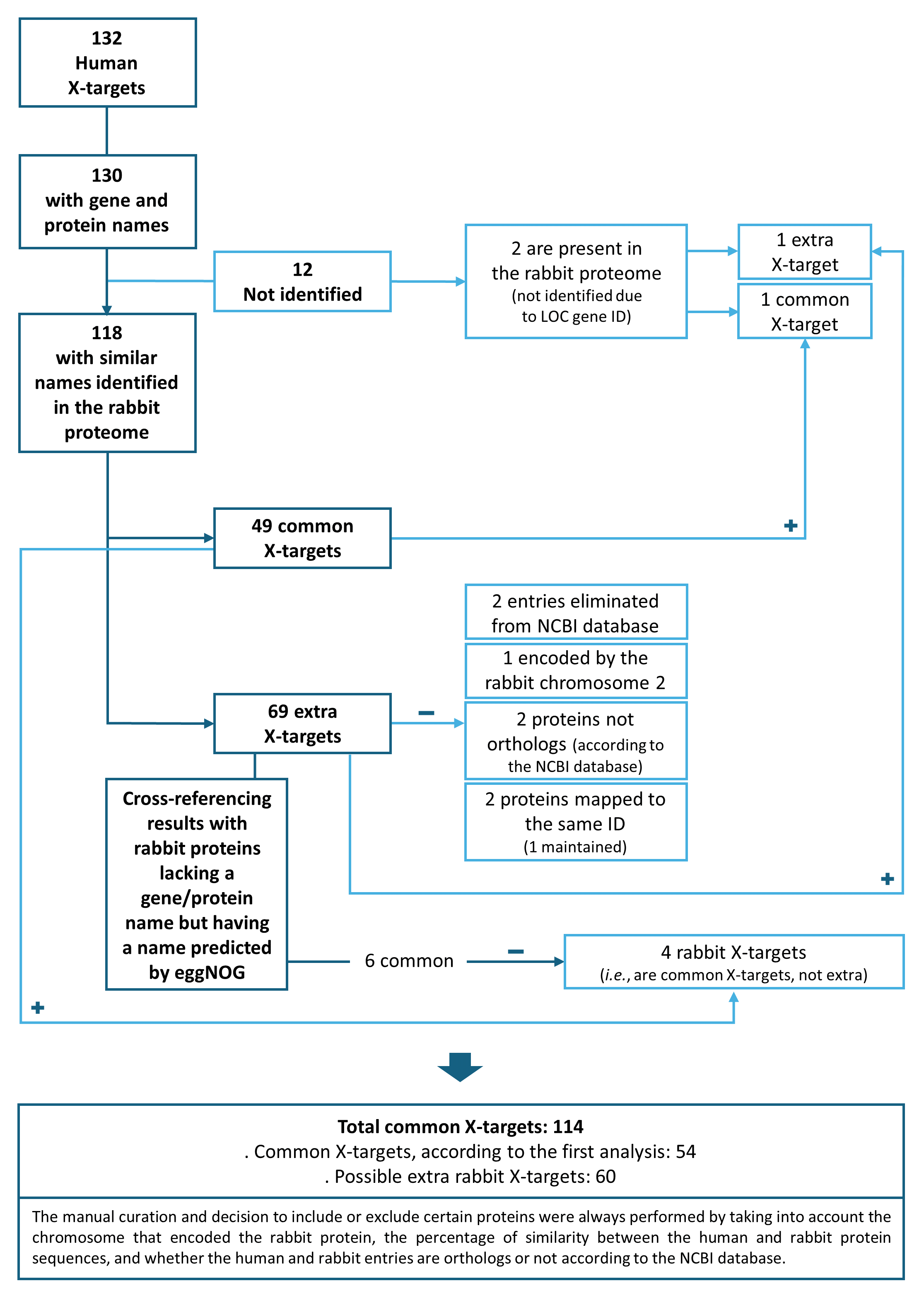

Supplement: Supplementary file 1 [file animals-14-00217-s001.zip › Figure S2.png]
